# Enhanced Aβ_1–40_ Production in Endothelial Cells Stimulated with Fibrillar Aβ_1–42_

**DOI:** 10.1371/journal.pone.0058194

**Published:** 2013-03-07

**Authors:** Jayakumar Rajadas, Wenchao Sun, Hai Li, Mohammed Inayathullah, Damiano Cereghetti, Aaron Tan, Valeria de Mello Coelho, Francis J. Chrest, John W. Kusiak, Wanli Wei Smith, Dennis Taub, Joseph C. Wu, Joseph M. Rifkind

**Affiliations:** 1 Molecular Dynamics Section, National Institute on Aging, Baltimore, Maryland, United States of America; 2 Laboratory of Immunology, National Institute on Aging, Baltimore, Maryland, United States of America; 3 Research Resources Branch, National Institute on Aging, Baltimore, Maryland, United States of America; 4 Biomaterials and Advanced Drug Delivery Laboratory, Stanford University School of Medicine, Stanford, California, United States of America; 5 Centre for Nanotechnology and Regenerative Medicine, UCL Division of Surgery and Interventional Science, UCL Medical School, University College London, United Kingdom; 6 Department of Medicine, Stanford University School of Medicine, Stanford, California, United States of America; 7 Institute for Stem Cell Biology and Regenerative Medicine, Stanford University School of Medicine, Stanford, California, United States of America; Oregon Health & Science University, United States of America

## Abstract

Amyloid accumulation in the brain of Alzheimer’s patients results from altered processing of the 39- to 43-amino acid amyloid β protein (Aβ). The mechanisms for the elevated amyloid (Aβ_1–42_) are considered to be over-expression of the amyloid precursor protein (APP), enhanced cleavage of APP to Aβ, and decreased clearance of Aβ from the central nervous system (CNS). We report herein studies of Aβ stimulated effects on endothelial cells. We observe an interesting and as yet unprecedented feedback effect involving Aβ_1–42_ fibril-induced synthesis of APP by Western blot analysis in the endothelial cell line Hep-1. We further observe an increase in the expression of Aβ_1–40_ by flow cytometry and fluorescence microscopy. This phenomenon is reproducible for cultures grown both in the presence and absence of serum. In the former case, flow cytometry reveals that Aβ_1–40_ accumulation is less pronounced than under serum-free conditions. Immunofluorescence staining further corroborates these observations. Cellular responses to fibrillar Aβ_1–42_ treatment involving eNOS upregulation and increased autophagy are also reported.

## Introduction

Alzheimer’s disease (AD) is characterized by neuronal degeneration and accumulation of senile plaques, which are composed of amyloid-β (Aβ) peptides predominantly consisting of 40 and 42 amino acids [1], [2]. The excessive accumulation of Aβ peptides in AD may be due to enhanced endoproteolytic cleavage of membrane bound amyloid precursor protein (APP), over-expression of APP and/or decreased clearance of Aβ from the central nervous system (CNS) [3]–[5].

Postmortem analyses of AD subjects reveal that amyloid plaques in the brain suffuse vascular cells in addition to the parenchymal. The implications of this vascular infiltration for AD has been less well studied than the parenchymal Aβ, but has generated considerable interest with studies that β-amyloid fibrils accumulate in small arteries, arterioles and capillaries of the brain [6]–[8]. Cerebrovascular amyloid toxicity generally manifests itself in the breach of the blood-brain-barrier and enhanced inflammation in the cerebrovasculature [9], [10].

The mechanism for the onset of pathological vascular changes has yet to be elucidated [11]. Two mechanisms that have been proposed involve: (1) The production of excess superoxide by amyloid-β induced oxidative stress [12], [13] and (2) the formation of amyloid aggregates whose resistance to protease degradation turns them into cellular “tombstones” that impair blood flow and cellular function [14], [15].

The oxidative stress mechanism was used to explain an *in vitro* investigation where the interaction with Aβ fibrils resulted in the endothelial lining of the rat aorta undergoing rapid damage leading to exposure of smooth muscle cells and connective tissue [16]. In agreement with these observations, it has been shown that antioxidant treatment and superoxide dismutase (SOD) treatment can reduce damage of endothelial cells caused by amyloid-β [17], [18].

The “tombstone” mechanism is consistent with biochemical and biophysical studies of synthetic Aβ peptides indicating that the more toxic Aβ peptides ending at residue 42 aggregate more rapidly than peptides of 39 or 40 amino acids [19]–[22]. This feature of Aβ_1–42_ makes it less susceptible to proteolytic degradation [23]–[25]. Further support for this mechanism comes from studies on the APP mutation found in HCHWA-Dutch type, which results in the production of Aβ with enhanced tendency to aggregate relative to that of wild type Aβ. Fibril formation in this mutation is limited to the cerebrovasculature and amyloidosis leads to cerebral hemorrhage [26], [27].

APP synthesis and processing to Aβ normally takes place only to a limited extent in endothelial cells [28]. It has, however been shown that amyloids can alter the expression pattern of specific proteins. Thus, the accumulation of Aβ_1–42_ in lysosomes down regulates the catabolism of APP resulting in enhanced production of amyloidogenic fragments [29]. Production of individual isoforms of Aβ induced by the same isoform has been shown in smooth muscle cells [30]. On the basis of these studies we investigated the possibility that amyloid fibrils can induce synthesis of more APP and amyloid of its other isoforms. Such a process would provide a synergistic mechanism whereby amyloid fibrils in circulation potentiate damage to the blood brain barrier endothelium. For this purpose we used synthetic, preformed Aβ_1–42_ fibrils and established the resultant accumulation of APP and Aβ_1–40._

## Materials and Methods

### Reagents

Media for cell culture was obtained from Invitrogen (Carlsbad, CA). Synthetic Aβ_1–40_, Aβ_1–42_ and monoclonal antibody against Aβ_1–40_ were purchased from Biosource International (Camarillo, CA). Fluorescein and phycoerythrin labeled streptavidin were purchased from PharMingen (Becton Dickinson, San Jose, CA.).

### Preparation of Amyloid Fibrils

Synthetic Aβ_1–42_ was disaggregated by pretreatment with trifluoroacetic acid (TFA) followed by treatment with trifluoroethanol (TFE) three times to remove traces of TFA. After each step, solvents were evaporated to form a film. Aβ_1–42_ fibrils were formed from disaggregated Aβ_1–42_ by following a known procedure [31]. Briefly, Aβ_1–42_ (250 µM) was dissolved in phosphate buffered saline, (PBS), pH 7.5, and polymerized in Eppendorf tubes (1.5 ml), at 37°C for 2 days. Newly formed fibrils were then separated from monomeric peptides by centrifugation at 4°C for 90 min at 15,000 rpm, using a Sorvall RC-5B centrifuge. The fibril pellet was re-suspended in PBS and used for *in vitro* studies [31], [32].

### Cell Culture and Flow Cytometry

Human endothelial (Hep-1) cell line is a kind gift from Dr. John Kusiak at the National Institute on Aging. This is an immortal human endothelial cell line derived from an adenocarcinoma of the liver [33]. Cells were grown in DMEM medium containing 10% fetal bovine serum (FBS Prime, Biofluids), 1×MEM non-essential amino acids, 2 mM L-glutamine, 100 units/ml penicillin, and 100 µg/ml streptomycin in a humidified atmosphere at 37°C in the presence of 5% CO_2_. 1×10^6^ cells were incubated with varying concentrations of Aβ_1–42_ fibrils in the presence or absence of FBS for 12 hrs. Cells were then harvested, washed with PBS, fixed in 2% glutaraldehyde, and probed with biotinylated monoclonal antibodies against Aβ_1–40_. This antibody detects low molecular weight Aβ_1–40_, but not fibrils. Cells were counter-labeled with 20 µg/ml phycoerythrin-streptavidin for 15 to 30 min. Labeled cells were analyzed on a FACScan flow cytometer (Becton Dickinson Immunocytometry Systems, San Jose CA) equipped with a 15 mW argon laser. Phycoerythrin labeled cells were excited with 488 nm light from the argon laser and emitted fluorescence was collected using the FL-2 (585/42) bands pass filter. A total of 20,000 events were analyzed for each sample. The amount of Aβ_1–40_ present in the endothelial cells was determined by mean fluorescence values. Light scatter signals were also collected in the forward and side scatter detectors. Experiments were repeated three times. The mean and standard error of the mean values were calculated.

### Fluorescence Microscopy

Cells grown on glass cover slips were incubated at 37°C for 12 hrs, and then washed with PBS. Cells with and without Aβ treatment were subsequently fixed in 2% glutaraldehyde for 10 min and washed with 100 mM glycine in PBS containing 2% bovine serum albumin. After washing, samples were incubated for 1 hr with a biotin-conjugated anti-Aβ_1–40_ antibody (1∶500 dilution) at room temperature, washed with PBS and stained with FITC-streptavidin conjugate. Immunofluorescence images were captured with an Axiovert S100 microscope (Zeiss, Munich, Germany). Images were recorded at the same exposure time using a digital SPOT camera (Diagnostic Instruments, Sterling Heights, MI).

### Western Blotting

Cells were lysed in 300 µl of 20 mM HEPES, pH 7.4, supplemented with 2 mM EGTA, 50 mM β-glycerol phosphate, 1% Triton X-100, 10% glycerol, 1 mM dithiothreitol (DTT), 1 mM phenylmethylsulfonyl fluoride, 10 µg/ml leupeptin, 10 µg/ml aprotinin, 1 mM Na_3_VO_4_, and 5 mM NaF. The resulting lysates were resolved on 4–15% SDS Tris-glycine gels (30 µg/lane) and transferred onto polyvinylidene difluoride membranes (Millipore, Bedford, MA). The membranes were blocked in TBST (10 mM Tris-HCl, pH 7.4, 150 mM NaCl, 0.1% Tween 20) containing 5% non-fat milk and then probed with the following primary antibodies: monoclonal mouse anti-human APP (8E5 Elan Pharmaceuticals; 1∶500 dilution), monoclonal mouse anti-human endothelial nitric oxide synthase (eNOS) (BD Biosciences; 1∶2500 dilution), monoclonal mouse anti-human nNOS (BD Biosciences; 1∶2500 dilution), rabbit polyclonal anti-caveolin 1 antibody (ab2910 abcam; 1∶2000 dilution) and rabbit polyclonal anti-LC3B antibody (ab48394 abcam; 1∶1000 dilution). Proteins were detected with HRP conjugated secondary antibodies and enhanced chemiluminescence (ECL) reagents (NEN Life Science, Boston, MA). The blot was quantitated by densitometric analysis and the difference between treated and control groups were analyzed by unpaired t test (tails = 2) using Excel software.

## Results

### APP Upregulation by Fibrillar Aβ_1–42_

**Figure 1:**
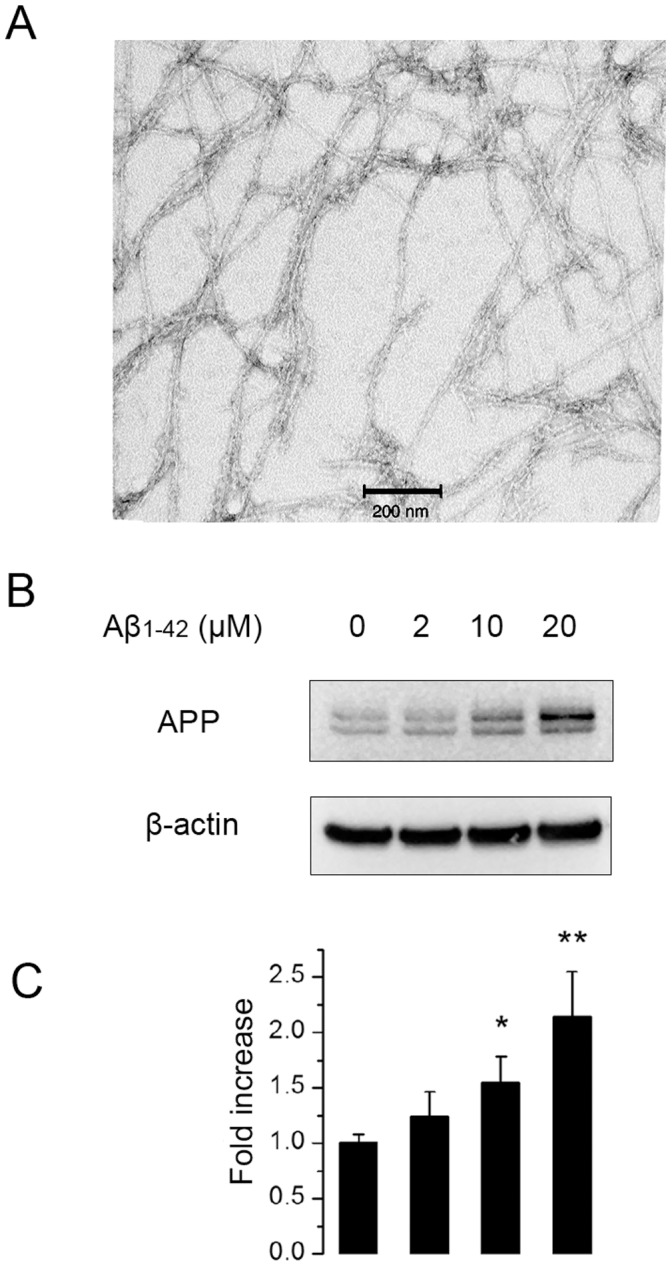
Fibrillar Aβ_1–42_ induced APP upregulation in Hep-1 cells. Morphology of fibrillar Aβ_1–42_ was confirmed by TEM (A).Cells were untreated or treated with 2 µM, 10 µM, 20 µM Aβ_1–42_ for 24 hrs. Cell lysates were subjected to Western blot analysis using 8E5 antibody (B). The blot was quantitated by densitometric analysis (C). The fold increase of APP production compared to untreated control cells were shown as mean ± SD (n = 4). *, P<0.05; **, P<0.01 compared to control.

Aβ_1–42_ fibrils were prepared from synthetic Aβ_1–42_ peptides and morphologically characterized by TEM (Fig. 1A). Western blotting was used to determine whether Aβ_1–42_ fibrils can increase synthesis of APP. For this purpose human endothelial cells (Hep-1) were incubated with Aβ_1–42_ fibrils for 12 hrs and then harvested in a lysis buffer for Western blotting. As shown in Fig. 1B and C, the amount of APP was increased in cells incubated with Aβ_1–42_ in a dose dependent manner.

### Aβ_1–40_ Production in Amyloidic Condition

The association of the increased APP synthesis with an increase in Aβ was investigated in the same cell model. It was possible to discriminate between newly synthesized amyloids and the Aβ_1–42_ used to treat Hep-1 cells by using antibodies that are specific for Aβ_1–40_, which only detects low molecular weight Aβ_1–40_, but not fibrils. The latter sequence accounts for about 90% of newly processed amyloid but no cross reactivity between Aβ_1–42_ and Aβ_1–40_ was observed, in agreement with previous reports [34].

**Figure 2:**
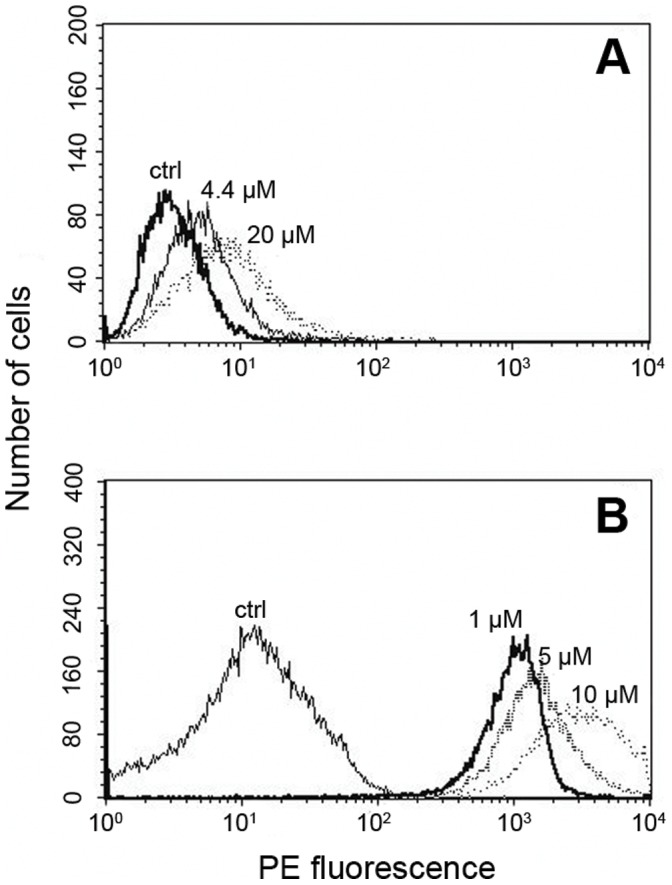
Detection of fibrillar Aβ_1–42_ induced Aβ_1–40_ production by flow cytometry. For the analysis of Aβ_1–40_ synthesis in amyloidic condition, Hep-1 cells were treated with fibrillar Aβ_1–42_ in the presence (A) or absence (B) of serum, stained with anti Aβ_1–40_ antibodies (biotinylated) and streptavidin conjugated phycoerythrin. Histograms are shown for the control with no added amyloid (ctrl) and for cells incubated with various concentrations of Aβ_1–42_.as indicated in the figure.

**Figure 3:**
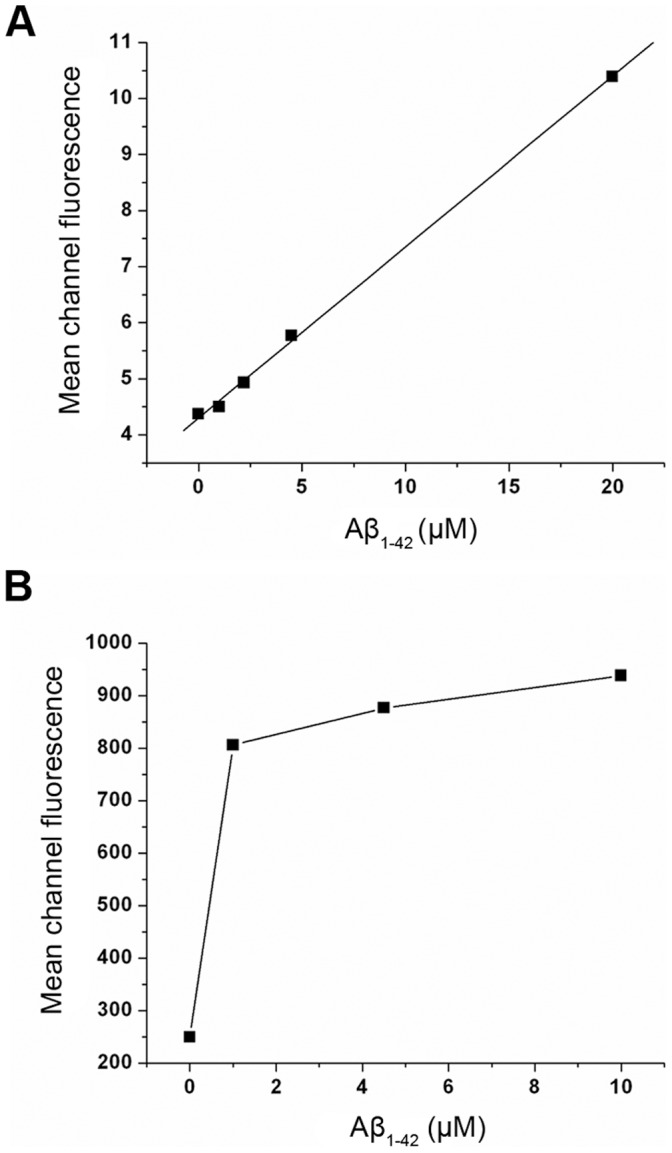
The mean fluorescence values obtained from the flow cytometry histogram were plotted against incubated Aβ_1–42_ concentration in the presence of serum (A) and under serum deprived condition (B).

The flow cytometry histograms, of cultures grown with and without FBS (Fig. 2A and B, respectively), show the amyloid-induced accumulation of Aβ_1–40_, which is further accentuated by the stress associated with the absence of serum. In both cases, it is clear that Aβ_1–42_ fibrils increase Aβ_1–40_ synthesis in a concentration dependent manner as shown by the plots of the mean fluorescence in Fig. 3. The concentration dependence further highlights the effect of stress associated with the absence of serum. Thus, in the presence of serum a gradual raise in fluorescence takes place over the range of amyloid concentrations tested (Fig. 3A), while more dramatic changes occur in the absence of serum (Fig. 3B) with the relative fluorescence increasing from 250 to 800 at the lowest concentration tested (1 µM). At higher concentrations only minor additional increases in fluorescence are detected.

Because the antibody used only detects low molecular weight amyloid, the observed Aβ_1–40_ cannot be derived from cleavage of added Aβ_1–42_ fibrils. To confirm that a cleaved fibril cannot be detected, Hep-1 endothelial cells were treated with 20 µM protofibrillar Aβ_1–40_ (10 hrs aged peptide) and analyzed by flow cytometry. Only a negligible increase in Aβ_1–40_ was observed and the fluorescence histogram was similar to that of control (data not shown).

### Cellular Morphology

**Figure 4:**
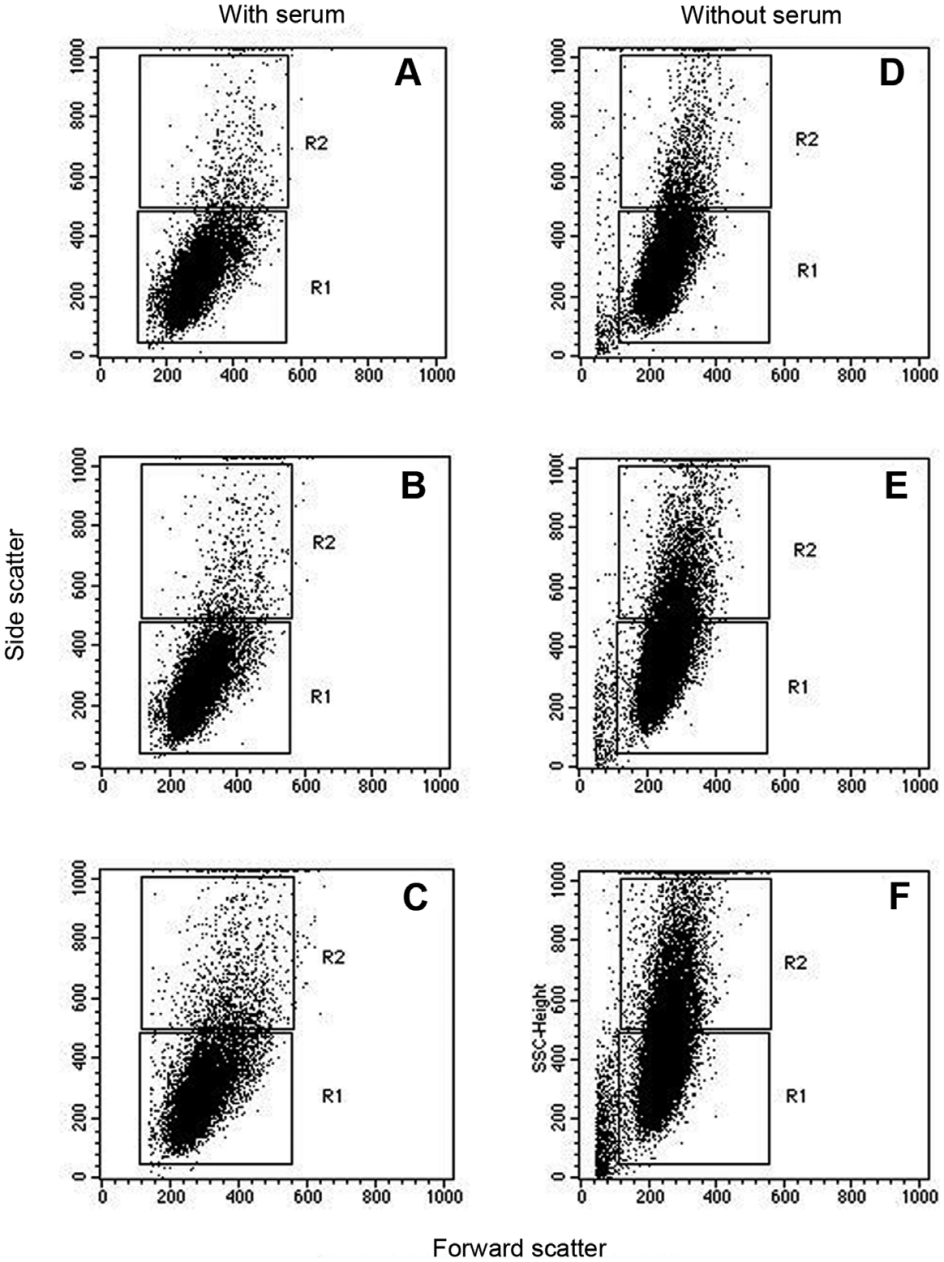
The morphological changes of Hep-1 cells incubated with Aβ_1–42_ in the presence and absence of serum analyzed by scattering data. The effect of Aβ_1–42_ concentration (A, D: 0 µM; B, E: 5 µM; C, F: 10 µM) in both forward and side scatter values is given in the dot plot.

Flow cytometry provides scattering data in addition to the fluorescence. The intensity light scattered at small angles (0.5–2.0°) from the incident laser beam (referred to as forward scattering) is proportional to the cell size. The side scattering at larger angles is a measure of structural changes such as granulated structure on the membrane or cytoplasm that increase the scattering. Examination of changes in scattering properties of Aβ_1–42_ treated cells revealed no difference in the forward scattering consistent indicating no change in cell size. On the other hand, changes in the granulated structure were apparent as evidenced by an increase in the intensity of side scattering (Fig. 4), which was even more pronounced in the absence of serum.

**Figure 5:**
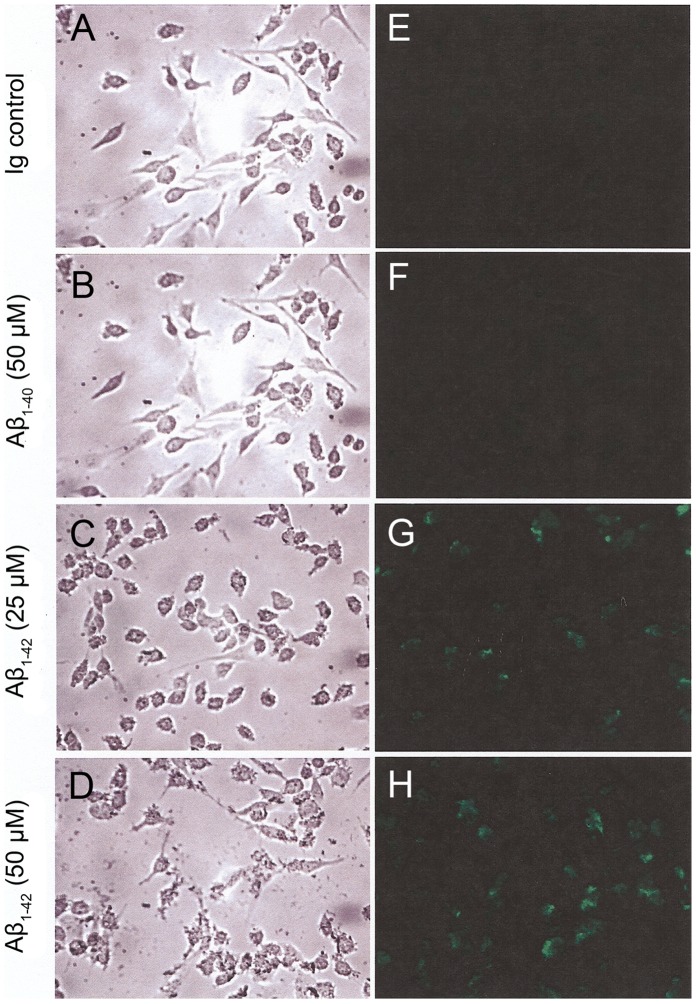
Aβ_1–40_ expression monitored by immunofluorescence microscopy. Endothelial cells were incubated with different concentrations of fibrils for 12 hrs and processed as described in the materials and method. Phase contrast (left panels) and fluorescence images (right panel) were taken showing the same field.

Morphological changes as well as increased production of Aβ_1–40_ in Aβ_1–42_ treated cells were further corroborated by light and confocal microscopy (Fig. 5). As found by flow cytometry, no effect was observed by microscopy when the endothelial cells were treated with Aβ_1–40_ instead of Aβ_1–42_ (Fig. 5A and B). In contrast, shape changes in some of the cells as well as numerous intracellular and superficial granular patches (tentatively attributed to big amyloid aggregates) were clearly visible by light microscopy when cells were treated with Aβ_1–42_ fibrils (Fig. 5C and D).

Treated and non-treated endothelial cells were stained with antibodies against Aβ_1–40_ and observed under confocal microscopy (Fig. 5E, F, G, H). A fluorescence signal was only detected in those cells that were exposed to Aβ_1–42_ (Fig. 5G and H).

### Aβ_1–42_ Treatment Affects eNOS Expression and Autophagy

**Figure 6:**
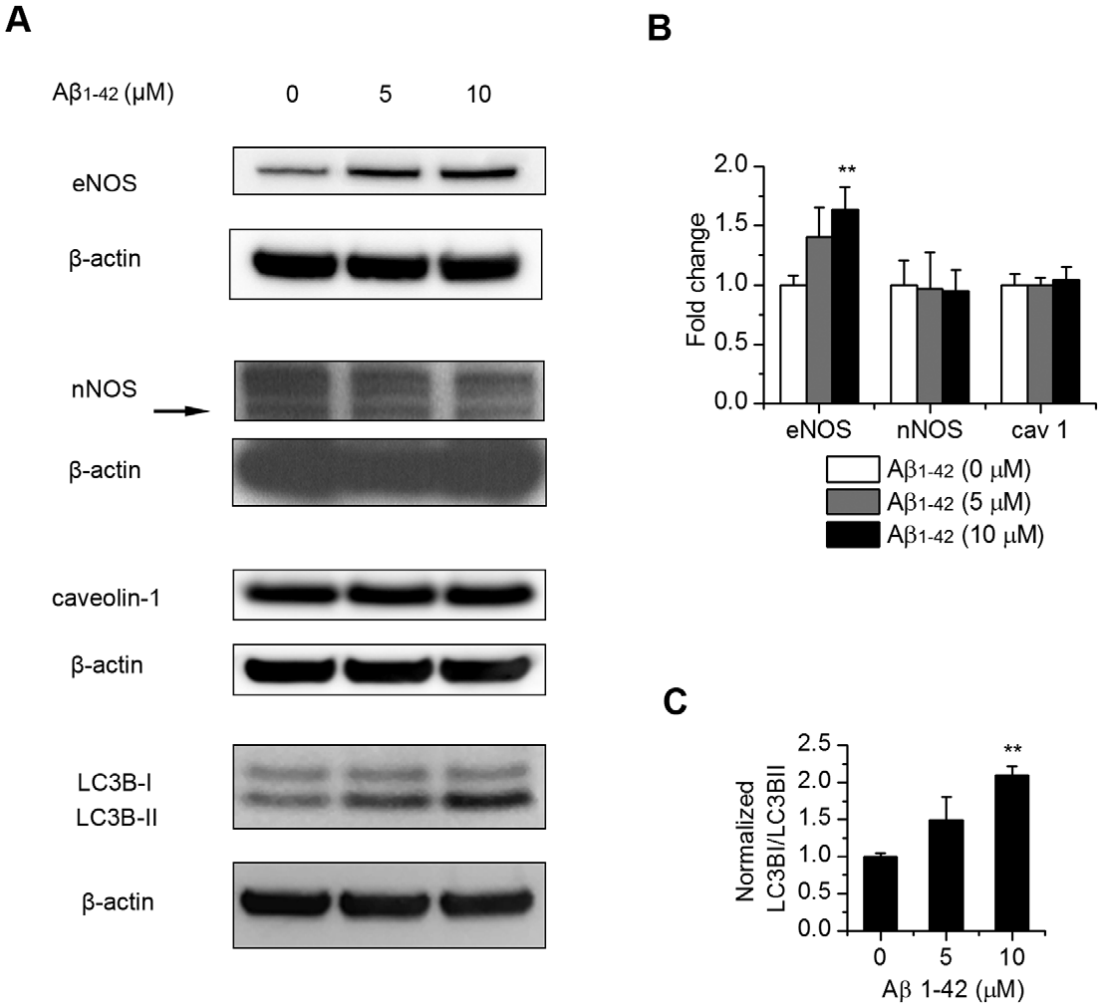
Affects of exogenous Aβ_1–42_ on the level of eNOS and autophagy in endothelial cells. Endothelial cells were treated with 5 µM and 10 µM Aβ_1–42_ and incubated for 12 hrs. Protein levels of eNOS, nNOS, caveolin-1 and the autophagy marker LC3B were analyzed by Western blotting (A). (B), quantitation of protein levels of eNOS, nNOS and caveolin-1 normalized to β-actin control. Results are mean ± SD (n = 3–5). (C), quantitation of LC3B II/I ratio normalized to β-actin control. Results are mean ± SD (n = 3). **, P<0.01 compared to control.

To further study the cellular response to Aβ_1–42_ treatment, we examined the protein levels of the nitric oxide synthases (NOS) which catalyze the production of NO, a known modulator of CNS vascular and neuronal function. After the cells were treated with 5 and 10 µM fibrillar Aβ_1–42_, Western blot of different NOS isoforms showed a mild but statistically significant increase of endothelial NOS (eNOS) and no change in neuronal NOS (nNOS) level (Fig. 6A and B). Inducible NOS (iNOS) was not detected with or without treatment (data not shown). Since caveolin-1 is shown to interact with eNOS within endothelial plasmalemmal caveolae and affect eNOS function [35], we also examined caveolin-1 protein level but found no change (Fig. 6A and B). Since autophagy is induced under conditions of cell stress and shown to be involved in APP processing [36], we studied the state of autophagy after Aβ_1–42_ treatment. Increased ratio of LC3B-II/I (Fig. 6A and C), a reliable marker of autophagosomes, indicated activation of autophagy.

## Discussion

Major attention has been devoted to the role played by neuronal cells in AD development. Most of the amyloid found in AD subjects is produced in the brain and contained in plaques with a high content of highly aggregated amyloids. However, cognitive function does not generally correlate with the level of cerebral plaques. It was only in recent years that the contribution of the cerebrovascular system has been appreciated [37]–[39]. In this respect, it should be noted that a large proportion of AD patients have cerebral amyloid angiopathy (CAA) involving vascular amyloidosis within the cerebral circulation. It has in fact been proposed that vascular pathology and the resultant impairment of oxygen delivery to the brain may play a primary role in the development of AD [40]. In this study, we have delineated a new role for the cerebrovascular system involving Aβ_1–42_ fibril induced APP synthesis in the human endothelial cell line Hep-1.

We first demonstrate that incubation of endothelial cells with fibrillar Aβ_1–42_ results in elevated levels of the APP (Fig. 1). Using an antibody specific for Aβ_1–40_, we also report an increase in the concentration of Aβ_1–40_ peptides. This finding is indicated both by flow cytometry (Fig. 2 and 3) and confocal microscopy (Fig. 5E, F, G, H). The increased levels of APP together with increased Aβ_1–40_ cannot be explained just by altered processing of APP and requires increased synthesis of APP by the endothelial cells.

Such an effect would seem to require that the Aβ_1–42_ fibrils are endocytosed by cells. Evidence for amyloid uptake and transcytosis in endothelial cells was provided more than a decade ago [41]. Among the potentially physiologically relevant amyloid receptors that can be involved in amyloid uptake, the RAGE (receptor for advanced glycation end products) [42]–[44] was shown to bind Aβ_1–40_ even in the fibrillar form with a high affinity [45], [46].

Support, from our data, for a contribution of stress to the observed increase in APP and Aβ comes from the augmentation of the effects under serum-deprived conditions [47]. An analogous stimulation of amyloid production has been reported [48] in serum-deprived human primary neuron cultures.

Altered cellular function can be triggered by the aggregation state of the amyloids in addition to stress. Our observation that only Aβ_1–42_ fibrils but not Aβ_1–40_ fibrils induce cellular changes implies that this phenomenon is triggered by the highly aggregated fibrillar form that is favored by the Aβ peptide ending at residue 42 [49]. Additional studies will be required to explain how the cellular changes produced by stress and/or amyloid aggregation induce APP synthesis and its processing. Two processes induced by exogenous Aβ fibrils are likely to be involved: inflammatory response and oxidative stress. As for increased APP synthesis, we have previously shown that cellular uptake of fibrillar Aβ induces interleukin-1α (IL-1α) expression [50]. The link between IL-1α and APP can be found in an earlier study which showed an IL-1α mediated upregulation of APP at the translational level [51]. Interestingly, the APP mRNA level was also unchanged after the Hep-1 cells were treated with Aβ_1–42_ (Rajadas unpublished data) indicating the increase of APP could also be translational in our study. As for increased Aβ production, there is ample evidence that in neuronal cells, Aβ accumulation and oxidative stress, each accelerating the other, generate a vicious circle of more Aβ production and oxidation [52]. Based on our observation, it is possible that the similar process is also taking place in endothelial cells. This notion is supported by an Aβ immunotherapy study which indicated that Aβ depositions in the brain parenchyma and blood vessels occur independently [53].

Once inside the cell, the amyloid can affect many other cellular responses. One of the cellular responses we observed is the upregulation of eNOS (Fig. 6) which is consistent with a previous study showing a Ca^2+^ dependent induction of eNOS by hydrogen peroxide in endothelial cells. This upregulation has been attributed to a compensatory mechanism for increased superoxide formation [54], [55]. Elevated eNOS expression has been observed in cardiovascular and other vascular pathologies, wherein increased levels of ROS have been detected [56]. Another cellular response we observed is increased autophagy (Fig. 6). Since autophagy is involved in APP processing as a protective mechanism [36], this response is expected and also explains the increased Aβ_1–40_ production. The intricate relations between oxidative stress and autophagy and its implication in AD vascular pathogenesis awaits further elucidation.

The increased cellular granulation indicated both by changes in side-scattering in the flow cytometry experiment (Fig. 4) as well as by microscopy (Fig. 5) seem to indicate damage to the cells. Thus, coupled with the altered cellular function, and the new synthesis of APP and its metabolism, amyloid toxicity results in damage to the endothelial cells.

In conclusion, our observation of increased APP and amyloid in ECs incubated with Aβ_1–42_ fibrils establishes, for the first time, that the exposure of endothelial cells to Aβ_1–42_ fibrils results in an elevated amyloid load in endothelial cells. This process provides a new potential pathway for amyloidosis with these newly formed amyloids in the endothelial cells able to contribute to both vascular amyloidosis and/or parenchyma amyloidosis.
